# Hyperthermic Intraoperative Thoracoabdominal Chemotherapy

**DOI:** 10.1155/2012/623417

**Published:** 2012-05-10

**Authors:** Paul H. Sugarbaker, David Chang, O. Anthony Stuart

**Affiliations:** ^1^Washington Hospital Center, Washington Cancer Institute, 106 Irving Street, NW, Suite 3900, Washington, DC 20010, USA; ^2^Westat, 1600 Research Boulevard, Rockville, MD 20850-3129, USA

## Abstract

Cytoreductive surgery combined with hyperthermic intraperitoneal chemotherapy (HIPEC) is a treatment option for selected patients with pseudomyxoma peritonei (PMP) and diffuse malignant peritoneal mesothelioma (DMPM). Tumor infiltration of the hemidiaphragm requiring partial resection occurs as a result of large volume and/or invasive disease at this anatomic site. Transmission of disease from abdomen to chest is a great danger in this group of patients. From a prospective database, patients who had diaphragm resection and then hyperthermic thoracoabdominal chemotherapy (HITAC) as a component of a cytoreductive surgical procedure were identified. Data from control patients receiving HIPEC or hyperthermic intrathoracic chemotherapy (HITOC) were analyzed for comparison. The morbidity, mortality, survival, and recurrence rate within the thoracic space were presented. Thirty patients had partial resection of a hemidiaphragm as part of a cytoreductive surgical procedure that utilized HITAC. The pharmacologic benefit of intracavitary chemotherapy administration was documented with an area under the curve ratio of intracavitary concentration times time to plasma concentration times time of 27 ± 10 for mitomycin C and 75 ± 26 for doxorubicin. Comparing percent chemotherapy absorbed for a ninety-minute treatment showed the largest for HIPEC, then for HITAC, and lowest for HITOC. The incidence of grade 3 and 4 adverse events was 43%. There was no mortality. Adjustments in the chemotherapy dose are not necessary with HITAC. The morbidity was high, the survival was acceptable, and intrathoracic recurrence was low.

## 1. Introduction

Increasing interest in the surgical management of peritoneal metastases from gastrointestinal cancer is evident from the many recent publications on this subject [1–5]. Improvements in surgical technology by using cytoreductive surgery with peritonectomy are a necessary part of these new management strategies [6]. Also, perioperative chemotherapy, especially hyperthermic intraperitoneal chemotherapy, has routinely been added to the surgical intervention [7]. Gastrointestinal cancer with peritoneal metastases may accumulate in large volume on the undersurfaces of the hemidiaphragms. Invasion of the hemidiaphragm, especially its tendinous midportion, may be required in order to achieve optimal cytoreduction. The perioperative chemotherapy and clinical management strategy for patients whose cytoreduction required partial excision of a hemidiaphragm are the subject of this paper. 

## 2. Materials and Methods

Permission to accumulate and analyze these data was obtained from the ethics committee of our institution. From a prospective database of patients with appendiceal mucinous neoplasms with peritoneal metastases, colon cancer patients with peritoneal metastases, gastric cancer with peritoneal metastases, and peritoneal mesothelioma patients; we identified those in whom a diaphragm resection was required at the time of cytoreductive surgery. The clinical features of these patients including their diagnosis, age, gender, and diaphragm resected (right versus left versus both right and left) were tabulated.

All of these patients had an attempt at complete removal of their peritoneal surface malignancy prior to the initiation of the hyperthermic intraoperative chemotherapy [8]. In all of these patients disease infiltration of the diaphragm required resection of a portion of the diaphragm in order to try and achieve the goal of complete cytoreduction. If even a small transection of the hemidiaphragm occurred, the entire central tendinous portion of the diaphragm was resected. The pleural space was visualized as possible through the opening in the hemidiaphragm. If cancer nodules within the thoracic space were visualized, one or several were biopsied. An attempt at pleurectomy was not added to the treatment strategy. This was to allow the free flow of hyperthermic intraoperative chemotherapy into the thoracic cavity from the abdominal cavity. To deliver the chemotherapy there was a single inflow catheter that was periodically moved around the abdominal space. There were three closed suction drains within the abdomen and a thoracostomy tube within the chest. The chest tube was 28 French diameter and many times larger than the intraabdominal drainage tubes thereby favoring heated chemotherapy flow from abdomen, through the diaphragm, and into the thoracic cavity.

During the chemotherapy treatment specimens from blood and the thoracoabdominal fluid were obtained at 15-minute intervals for 60 minutes and then a single sample at 90 minutes. These samples were centrifuged to remove debris or red blood cells. The cell-free solutions were frozen and stored for high-performance liquid chromatography (HPLC) analysis which was performed within 1 week.

The dose of chemotherapy was 15 mg/m^2^ for mitomycin C and 15 mg/m^2^ for doxorubicin. The volume of chemotherapy solution was always 1.5 liters/m^2^ of body surface. This was a volume of chemotherapy solution that would fill the peritoneal space and the thoracic space at the initiation of the combined thoracoabdominal chemotherapy lavage.

Following the hyperthermic thoracoabdominal chemotherapy treatments, all chemotherapy fluid was removed from the abdomen and pelvis. The volume of chemotherapy solution was carefully measured so that the total milligrams of chemotherapy that left the thoracoabdominal space into the body compartment could be calculated.

Following the chemotherapy treatments, the diaphragm was closed in a routine fashion with interrupted and continuous sutures. Thereafter, reconstruction of the gastrointestinal tract and closure of the abdomen occurred.

Pharmacokinetic studies of mitomycin C and doxorubicin were performed on peritoneal fluid and plasma in order to determine the relative exposure (area under the curve ratio) of chemotherapy in the peritoneal and pleural fluid versus chemotherapy in the plasma. The drug concentrations in these fluids were determined by HPLC assay as previously described [9, 10]. Also, 25 patients in whom the regional chemotherapy was confined to the peritoneal cavity (HIPEC) matched for age, diagnosis, and extent of disease (except for diaphragm resection) who had mitomycin C and doxorubicin pharmacokinetics determined were used as a comparison for data from patients who had both thoracic and abdominal chemotherapy lavage (HITAC). In a third group of patients, the hyperthermic chemotherapy treatment was limited to the thoracic cavity after pleurectomy and decortication (HITOC). The percent of the chemotherapy absorbed in these three groups of patients was compared.

### 2.1. Statistics

A Student's *t*-test (Microsoft Excel 2007) was used to compare the percent of total intracavitary chemotherapy absorbed over 90 minutes from the thoracoabdominal space versus the thoracic space. Survival analyses were prepared using the Kaplan-Meier method.

A postoperative prospective morbidity/mortality database was maintained on these patients. There were 8 categories of events and 47 items scored in the eight categories as previously described [11]. Also, followup on these patients in terms of long-term survival and recurrence of disease within the hemithorax was determined.

## 3. Results

There were a total of 30 patients with peritoneal metastases between January 2000 and September 2011 whose cytoreductive surgery required a diaphragm excision. Sixteen patients had appendiceal mucinous neoplasm, 5 colon cancer, 8 peritoneal mesothelioma, and 1 gastric cancer. The median age of these patients was 50 with a range of 33 to 68 years. There were 16 female patients and 14 male patients. The right pleural space was entered in 23 patients, the left pleural space was entered in 6 patients, and both right and left hemidiaphragms partially resection in a single patient. At the close of the cytoreductive surgery, a CC-0/CC-1 (complete) cytoreduction was recorded in 18 patients and a CC-2/CC-3 (incomplete) cytoreduction in 12 (Table 1). In 6 patients the cytoreduction was scored as incomplete because of residual disease within the thorax.

In the 25 control patients with hyperthermic chemotherapy limited to the peritoneal space, 23 had a diagnosis of appendiceal mucinous neoplasm, and two a diagnosis of colon cancer. In the three patients who received intrathoracic mitomycin C, all had an appendiceal mucinous neoplasm. In the four patients who received intrathoracic doxorubicin, three had appendiceal mucinous neoplasm and one had peritoneal mesothelioma (Table 1).

### 3.1. Pharmacokinetics of Hyperthermic Chemotherapy in Thoracic and Abdominal Cavity (HITAC)

**Figure 1:**
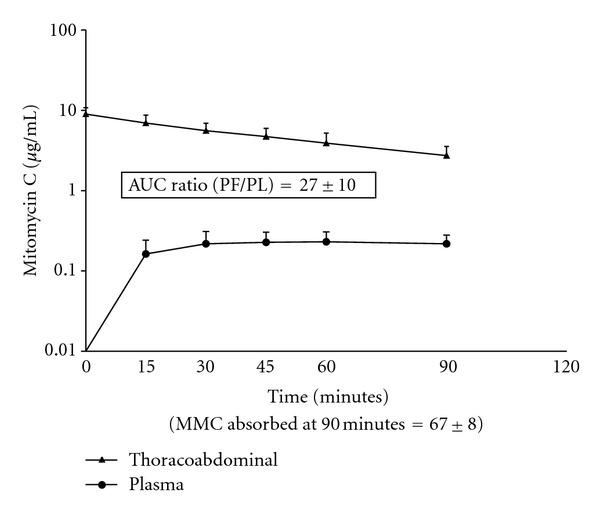
Pharmacokinetics of mitomycin C in 12 patients with simultaneous intraabdominal and intrathoracic chemotherapy used in conjunction with cytoreductive surgery.

In 12 patients complete pharmacokinetic data was available so that the mitomycin C concentrations in fluid from the thoracic and abdominal space could be determined along with plasma concentrations of this drug.  Figure 1 shows the area under the curve for thoracoabdominal mitomycin C as compared to the area under curve for plasma mitomycin C.The area under the curve ratio was 27 ± 10. Nine of these patients had appendiceal neoplasms, 2 colon cancer, and 1 peritoneal mesothelioma.

**Figure 2:**
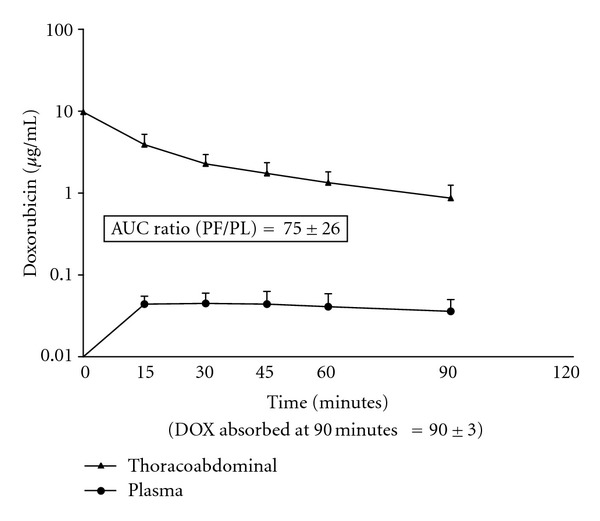
Pharmacokinetics of doxorubicin in 12 patients with simultaneous intraabdominal and intrathoracic chemotherapy administration in conjunction with cytoreductive surgery.

Similar data was obtained from the same 12 patients who received intrathoracic and abdominal doxorubicin. The area under the curve for thoracoabdominal fluid as compared to the area under the curve for plasma is shown in Figure 2. The area under the curve ratio was 75 ± 26.

### 3.2. Comparison of Thoracic and Abdominal Chemotherapy Treatment (HITAC) to a Chemotherapy Lavage Limited to the Abdominal Space (HIPEC) and to a Chemotherapy Lavage Limited to the Thoracic Space (HITOC)

**Figure 3:**
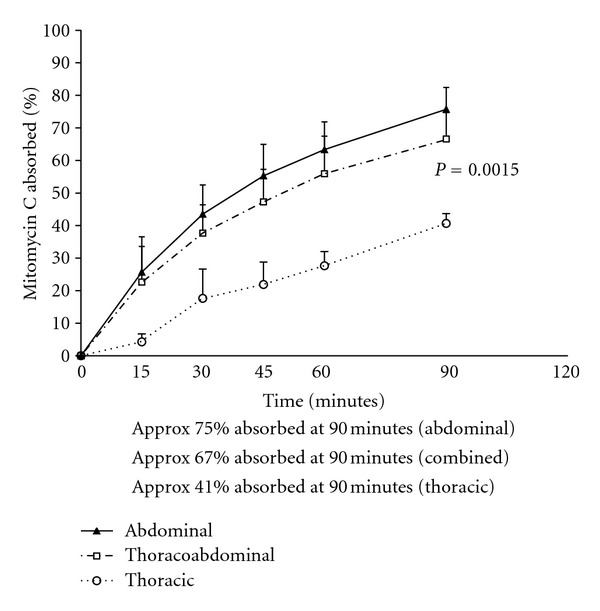
Comparison of percent mitomycin C absorbed after 90 minutes of treatment from the abdominal cavity, combined thoracic and abdominal cavity, and the thoracic cavity alone.

**Figure 4:**
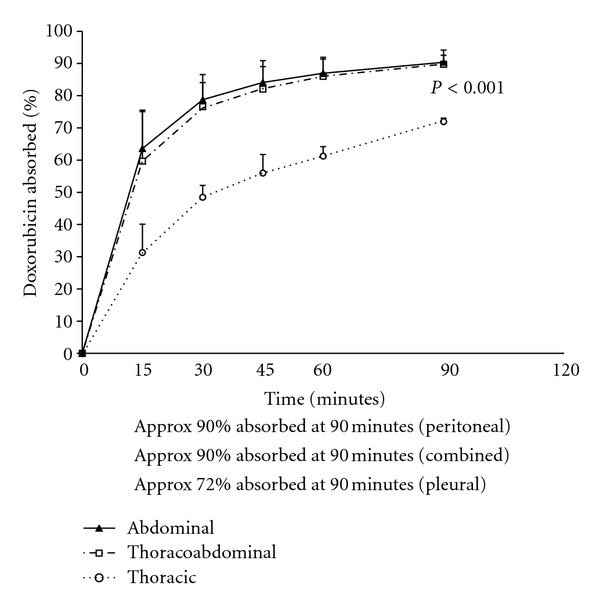
Comparison of percent of doxorubicin absorbed after 90 minutes of treatment from the abdominal cavity, thoracic and abdominal cavity, and the thoracic cavity alone.

In 25 control patients in whom hyperthermic intraoperative chemotherapy treatment was limited to the abdominal and pelvic space (HIPEC), the percent of drug absorbed from the peritoneal cavity over the 90 minutes of treatment was determined. Also in three patients the hyperthermic mitomycin C treatments were limited to the thoracic cavity (HITOC). These results were compared to the percent of chemotherapy absorbed in patients with thoracic and abdominal chemotherapy lavage (HITAC).Figure 3 shows the results with mitomycin C. In patients with HIPEC mitomycin C treatment, 75% of the total drug administered at time zero was absorbed at 90 minutes. In patients with HITAC 67% was absorbed. In the patients with HITOC, only 41% was absorbed from this space at 90 minutes. This was highly significant as compared to the HITAC (*P* = .0015).  Figure 4 shows similar data for doxorubicin. Very similar percent absorption over 90 minutes occurred in patients receiving HIPEC as compared to HITAC. For HITOC with doxorubicin, 72% was absorbed. This was statistically different when compared to the HITAC (*P* < .001).

### 3.3. Prospective Morbidity and Mortality Data

The prospective morbidity/mortality database on this group of 30 patients showed that no deaths occurred. In 11 of these 30 patients (37%) at least one grade III adverse event was recorded. Also in 8, at least one grade IV adverse event occurred (27%). The combined incidence of grade III and IV events was 43%. There was a single adverse grade III or IV event in 3 patients. However, most patients who had one adverse event experienced others. Five had two adverse grade III or IV events, one had three adverse events, one had 4 adverse events, and one had 7 adverse events recorded. The 30 adverse events recorded in 13 patients are listed in Table 2.

### 3.4. Survival Data

**Figure 5:**
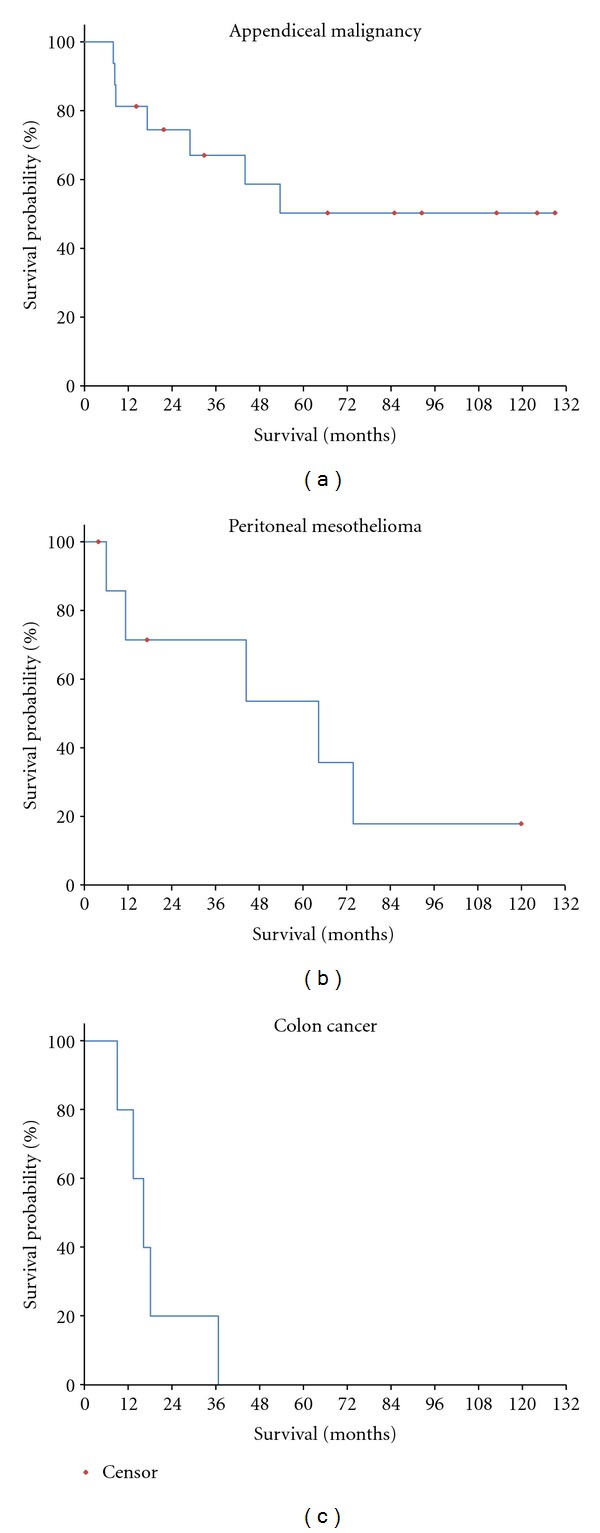
Survival for 29 patients who had a resection of the diaphragm as part of the cytoreductive surgical procedure combined with hyperthermic intraoperative thoracoabdominal chemotherapy (HITAC). (a) appendiceal malignancy (*N* = 16), (b) peritoneal mesothelioma (*N* = 8), and (c) colon cancer (*N* = 5).

The overall survival of these groups of patients is presented in Figure 5. Median survival for appendiceal malignancy patients was 129 months, for peritoneal mesothelioma patients 44.6 months, and for colon cancer patients 13.4 months. In the analysis of the appendiceal mucinous neoplasm patients, adenomucinosis histology (one patient) was combined with those patients with peritoneal mucinous carcinoma (15 patients). The single gastric cancer patient died at 5 months.

### 3.5. Recurrence

All patients have been followed to determine if cancer progressed within the thoracic cavity after HITAP. A single patient with appendiceal malignancy who had both the right and left thoracic space entered at the time of cytoreduction recurred within the pleural space. Two mesothelioma patients recurred within the pleural space.

## 4. Discussion

### 4.1. Pleural Disease Progression in Patients with and without Hyperthermic Intraoperative Thoracoabdominal Chemotherapy (HITAC)

In a previous publication looking at patterns of failure in the treatment of patients with pseudomyxoma peritonei, our group reported 6 of 8 patients to develop disease within the ipsilateral thorax if the pleural space was entered as a result of a subdiaphragmatic peritonectomy [12]. In these patients the diaphragm was closed prior to the intraperitoneal chemotherapy treatment. Therefore, little if any direct contact of chemotherapy solution with the pleural space could occur. In these eight patients, 75% had iatrogenic dissemination of disease from abdominal space to thoracic cavity as a result of an interruption of the integrity of the hemidiaphragm. In the 118 patients in Zoetmulder's manuscript, pleural dissemination did not occur unless the pleural space was entered. These patients all received early postoperative intraperitoneal chemotherapy (EPIC). In contrast, we have followed-up all 16 appendiceal malignancy patients treated with HITAC for disease progression within the pleural space. Pleural progression occurred in a single patient. This was the patient who required both right and left diaphragm resection as the time of her primary cytoreduction. These data strongly suggest that HITAC is an essential part of the treatment of peritoneal metastases if diaphragm resection is required.

### 4.2. Abdominal versus Thoracic and Abdominal versus Thoracic Chemotherapy Lavage

The pharmacokinetic advantage of chemotherapy administration into the peritoneal cavity has been described in many prior publications. Van der Speeten and colleagues presented doxorubicin levels in plasma, peritoneal fluid, and tumor nodules. The ratio of drug concentration within the peritoneal space as compared to that present in the plasma was 73 times greater within the peritoneal fluid. Doxorubicin levels within tumor tissue were 1.8 times higher than in the peritoneal fluid [13]. Likewise, pharmacokinetic data regarding intraperitoneal administration of mitomycin C has been reported in the past. Van der Speeten and colleagues showed that the exposure of peritoneal surfaces to be 26 times higher than exposure within the plasma [9]. Technical difficulties with extracting mitomycin C from body tissues precluded the data regarding tissue levels of mitomycin C [9]. Because of the large increase in total diffusion surface when the pleural space is added to the abdominal space for HITAC, we expected a more rapid clearance of the intracavitary chemotherapy [14].

To our surprise adding the pleural space to the HIPEC procedure did little to change the pharmacokinetics of the chemotherapy. This is important in that the percent of chemotherapy absorbed from the thoracic and abdominal space should predict the likelihood of hematologic toxicity in this group of patients. Apparently, the absorption of chemotherapy from the pleural space through the parietal pleura and visceral pleura is considerably less efficient than from the abdominal and pelvic cavity. Data showed the clearance of mitomycin C over 90 minutes to be approximately 75% from the abdominal space, 67% from the thoracoabdominal space, and only 41% when the lavage is limited to the pleural space. Also, for doxorubicin, 90% of the drug was absorbed from the abdominal space, 90% from the thoracoabdominal space, and only 72% absorbed from the pleural space. This lack of permeability of the pleura to cancer chemotherapy accounts for the similarities of drug absorption from abdominal space as compared to thoracoabdominal space despite a large increase in the total diffusion surface.

This observation is important in this group of patients. It indicates to us that the dose of chemotherapy that would be used to treat the abdomen and pelvis can be estimated to be the same dose as that to treat the thorax and abdominal space. No adjustments in the standardized HIPEC chemotherapy orders are necessary in this clinical situation.

### 4.3. Adverse Events with Diaphragm Resection

In this group of patients the resection of the hemidiaphragm was associated with a 43% incidence of grade III or IV adverse events. There was no mortality. Our most recent morbidity data showed a 0.7% mortality, a grade III incidence of 20% and grade IV of 12% with cytoreductive surgery limited to the abdomen and pelvis [15]. Our data in 30 patients with diaphragm resection, chemotherapy lavage of both thorax and abdomen, and then a suture repair of the diaphragm showed no mortality, a grade III incidence of 37%, and a grade IV of 27%. Apparently, the subdiaphragmatic peritonectomy with partial excision of a hemidiaphragm identifies a group of patients with extensive cytoreduction and a greater likelihood of morbidity. However, we think these data show that cytoreductive surgeons should not hesitate to perform a resection of the tendinous midportion of the hemidiaphragm in order to achieve a CC-0/CC-1 cytoreduction and combine this surgery with HITAP. Benefits are expected in terms of local control using this approach.

### 4.4. Survival of Patients Having Diaphragm Resection

Figure 5 shows the survival with diaphragm resection as a part of a cytoreductive surgical procedure. The appendiceal malignancy patients showed the longest median survival (129 months). Also, a median survival of 45 months for peritoneal mesothelioma patients is acceptable. One of the five colon cancer patients who required diaphragm resection for a complete cytoreduction was a long-term survivor.

### 4.5. Current Recommendations for Management of Diaphragm Resection

Our current recommendation for management of diaphragm whose central tendon is infiltrated by tumor is as follows. If during the right or left subdiaphragmatic peritonectomy it becomes clear that partial resection of the diaphragm is necessary, the dissection beneath the diaphragm ceases and the cytoreduction moves to a different part of the abdomen and pelvis. The cytoreduction is completed as thoroughly as possible at all other sites within the abdomen and pelvis with the exclusion of the hemidiaphragm. Then, there is a vigorous six liter irrigation of the entire abdomen and pelvis in order to mechanically clear free cancer cells from the abdomen and pelvis. Alternatively, the cytoreduction may turn out to be incomplete so that diaphragm resection is not required to finish the CC-2/CC-3 cytoreduction.

After this mechanical cleansing of the abdomen and pelvis of cancer cells, the required resection of the hemidiaphragm occurs. The specimen is carefully labeled in terms of its abdominal and thoracic orientation. It is important to determine if the disease has invaded full thickness through the hemidiaphragm. The thoracic cavity is carefully inspected for nodules of mucinous cancer or mesothelioma. Nodules that can be excised without extensive pleurectomy are removed and also submitted for permanent histopathologic study. Abdominal drains and an inflow catheter are placed. A thoracostomy tube is placed within the chest cavity. The three abdominal drains and the thoracostomy tube are simultaneously used for drainage of the chemotherapy. The inflow catheter is sequentially placed in the pelvis, the right paracolic sulcus, the left paracolic sulcus, under the intact hemidiaphragm, and finally through the diaphragm into the chest. After the chemotherapy lavage is complete the fluid is suctioned from the abdomen and pelvis. A count of all laparotomy pads is obtained and then the chest cavity is closed. A series of interrupted and running #1 Vicryl sutures (Ethicon, Cincinnati, OH) are used. The use of a prosthetic diaphragm patch is seldom, if ever, necessary.

### 4.6. Open versus Closed Hyperthermic Intraperitoneal Chemotherapy

Currently, there remains some controversy regarding the methodology for administration of hyperthermic intraperitoneal chemotherapy. In the closed technique there is less danger, theoretically, for aerosol contamination of the operating theater. With the closed technique, if the diaphragm was opened as a result of cancer infiltration, this opening would be closed prior to the initiation of HIPEC. This is a theoretical disadvantage in that tumor cells can only be mechanically removed from the thoracic cavity rather than treated with the chemotherapy. In the open methodology the combined abdomen and chest cavities are simultaneously lavaged with the chemotherapy solution for the full 90 minutes. Data from this paper suggests that the administration of HITAC does not require dose adjustments of the chemotherapy as compared to HIPEC. Also, these data suggest that local control as a result of HITAC within the thoracic space is excellent. Following the hyperthermic chemotherapy lavage of the contaminated pleural surfaces, the diaphragm would be closed leaving a large bore thoracostomy tube within the thoracic cavity. A separate inflow catheter into the chest was not necessary because of the large volume of chemotherapy outflow through the open diaphragm and out through the thoracostomy tube. A theoretical and probably actual clinical advantage of the open method over the closed method occurs in patients required to have diaphragm resection.

### 4.7. Rationale for HITAC in Patients with CC-2/CC-3 Cytoreduction

The data regarding palliative benefit of HIPEC in patients with CC-2/CC-3 cytoreduction has never been rigorously studied. There is no doubt that incomplete cytoreduction is associated with a poor prognosis in appendiceal cancer, colorectal cancer, and peritoneal mesothelioma patients. However, when added to an extensive debulking procedure, the hyperthermic intracavitary chemotherapy may achieve a partial response and prolong the patient's life. This may be most likely if all adhesions on bowel loops are separated so the HIPEC is in contact with all peritoneal surfaces. Also, any patients who have ascites as a component of their peritoneal surface malignancy should receive intracavitary chemotherapy in order to guard against debilitating ascites occurring as the disease progresses. Garofalo and Valle showed that HIPEC is an excellent treatment for the management of cancerous ascites [16].

## 5. Conclusions

HITAC is a treatment option for cytoreductive surgery in appendiceal and DMPM patients when the diaphragm must be partially resected as part of a cytoreductive surgical procedure. The pharmacokinetic advantage of direct intracavitary administration is preserved when HITAC is utilized. Also, judgments in the chemotherapy dose were not found to be necessary with HITAC as compared to HIPEC. An assessment of adverse events showed a 43% incidence of grade III or grade IV adverse events which is higher than reported for most groups of patients undergoing cytoreductive surgery and HIPEC. However, the survival was acceptable and the incidence of intrathoracic recurrence was low.

## Figures and Tables

**Table 1:** Clinical and demographic information on patients having hyperthermic intraoperative thoracoabdominal chemotherapy (HITAC), patients having hyperthermic perioperative chemotherapy (HIPEC), and patients having hyperthermic intrathoracic chemotherapy (HITOC).

DEMOGRAPHICS	HITAC	HIPEC	HITOC
Total patients	30	25	5
Male	14	10	1
Female	16	15	4
Diagnosis			
Appendix	16	23	4
Colon	5	2	
Peritoneal mesothelioma	8		1
Gastric	1		
Right	23	Not applicable	3
Left	6	Not applicable	2
Right and left	1	Not applicable	0
Complete cytoreduction			
CC-0/CC-1	18	21	5
Incomplete cytoreduction			
CC-2/CC-3	12	4	0
Median age	50	48	50
Range	33–68	33–67	43–58

**Table 2:** Causes of grade III or IV adverse events. There were 30 adverse events in 13 patients (43%).

	III	IV	III + IV (%)
Line sepsis	4	0	4 (13)
Anemia (Hgb < 6.5)	3	0	3 (10)
Pneumonia	0	2	2 (7)
Venous thrombosis	2	0	2 (7)
Anastomotic leak	0	1	1 (3)
Intraabdominal abscess	0	1	1 (3)
Urinary tract infection	3	0	3 (10)
Profound neutropenia (WBC < 1000)	2	1	3 (10)
Hartmann stump leak	0	1	1 (3)
Unscheduled hospital readmission	1	1	2 (7)
Pleural effusion requiring thoracentesis	2	0	2 (7)
Postoperative bleed requiring reoperation	0	3	3 (10)
Renal failure requiring hemodialysis	0	1	1 (3)
Respiratory failure requiring tracheostomy	0	1	1 (3)
Bile leak from liver surface requiring reoperation	0	1	1 (3)

Total	17	13	30 (43)
